# Alumni Association Medical Department of the University of Buffalo

**Published:** 1913-07

**Authors:** 


### Alumni Association Medical Department of the University of Buffalo

The thirty-eighth annual meeting took place May 27-30. The first day (Tuesday) was given up to registration and the afternoon and evening to class reunions. The class of 1873, in charge of Dr. William H. Slacer, held its dinner at the Roanoke Hotel. Dr. Charles G. Stockton entertained his classmates of '78 at the Saturn Club. The class of 1883 had an auto ride in the afternoon, a dinner at the Buffalo Club, followed by a theater party at the Teck Theater. Classes of '93, 1903 and 1908 held their respective class dinners at the Hotel Statler, while the class of '98 dined at the Castle Inn, the arrangements being made by Irving R. Johnson. The dinners were well attended and there was a marked expression of loyalty to the University in the toasts rendered.

### Scientific Program.

The scientific feature of the program consisted of papers given on the morning and afternoon of Wednesday and Thursday. The contributions were all of a high class scientific character, and were listened to with much interest by the alumni present. On Wednesday morning Doctor Frederick H. Pratt, the newly elected Professor of Physiology, gave a paper entitled "The Effects of Carbon Dioxide on the Respiratory Center," which was followed by a demonstration in the Physiological Laboratory at 12.30.

Dr. Pratt's paper was introductory to a demonstration of the aero-plethysmograph, which took place at noon in the laboratory, together with a demonstration of various methods of laboratory teaching. The preliminary remarks were devoted to a statement of the problem basic to the theory of ventilation—the question as to the importance of the chemical purity of the air as contrasted with that of humidity and temperature conditions. He explained the difference in result following immersing of the body in respiratory products, pure air being breathed, and that following breathing of respired air, the body immersed in pure air. These two conditions were pointed out as the main divisions of this important problem.

The demonstration later showed how markedly the chemical purity of the respired air might diminish without any of the supposedly toxic conditions accompanying such procedure. With the aero-plethysmograph the subject was able to rebreathe his own alveolar air until stimulation of the respiratory center doubled the depth of respiration—a condition wholly beyond comparison with that in the worst ventilated room. The air may cease to support combustion long before sensations of discomfort are felt, provided the skin surface is bathed in freely moving air of low humidity. The marked influence of the heightened carbon dioxide tension on the respiratory center, and experiments testing reflex efficiency under such conditions were explained.

Students' laboratory notes were on exhibition, and demonstrations by first year students were carried on under the head of "A New Method of Teaching Heat Regulation," and "Blood Pressure Changes Under Vagus and Depressor Stimulation." These demonstrations were intended as typical examples of the laboratory teaching methods now adopted in the school—the student learning through his own performance of experimental work.

The second paper, entitled "The Problem of Selective Action" (some laboratory methods in Pharmacology), was given by Dr. John L. Butsch, who came to us last year to take charge of the Department of Pharmacology. This paper was more fully amplified in the laboratory demonstrations given by Professor Butsch, assisted by his students, on Thursday noon. There were four tables arranged to demonstrate the methods of teaching used during the past year to show the action of drugs in the regular course for students. On table No. 1 there was a demonstration of the general protoplasmic poisons on cells—the types of drugs used were quinine, strychnine and cocaine. The cells used for demonstrative purposes were yeast cells and the paramecium. The yeast cells were suspended in a 5 per cent. glucose solution mixed with the proper concentration of the drugs, then placed in a Smith Fermentation tube and kept at the body temperature (37° centigrade) for half an hour, and the retardation of fermentation noted. A hanging drop was made of the paramecium and a drop of each blood in solution mixed with it on a separate slide. The action of the drug on the single celled organism was then carefully watched through the microscope.

On table No. 2 an animal was arranged for circulatory and respiratory observations, to show the relative efficiency of the various methods of drug administration, also the action of drugs on the circulatory and respiratory systems. The graded effects upon the height of blood pressure in the carotid artery was shown by using adrenalin in ¼ c.c. doses—the least effect being obtained from the subcutaneous injection, more by the intramuscular and the greatest effect by the intravenous method. The effect of administering drugs by inhalation was in the first instance shown by ether narcosis and secondly the vascular effects produced by amyl nitrite. Later the action of digitalis on a circulatory apparatus was demonstrated. The toxic action of carbolic acid after absorption and the rapidity of the restoration of the animal to normal condition with the intravenous injection of 2 per cent. solution of sodium sulphate was then shown.

On table No. 3 there was a demonstration of stimulation and depression, the action of poisons, the treatment of poisons, the method of locating the exact point of the action of a drug on the neuro-muscular apparatus, racial idiosyncrasy, physiological antidotes and chemical antidotes.

On table No. 4 there was a demonstration of the method of perfusion of a cold-blooded heart and the ion action of potassium, sodium and calcium. Also the therapeutic and toxic action of digitalis on cardiac muscle. Other demonstrations comprised the action of drugs in the blood, the action of locally acting emetics and centrally acting emetics. Defibrinated beef blood was used and a demonstration of the action of carbon monoxide, potassium chlorate, potassium ferrocyanide, sodium nitrite by means of specroscope.

Dr. Robert P. Kennedy, Instructor in Chemistry, gave a short talk followed by demonstration on Chemotaxis, showing how an extra molecule of arsenic could be inserted in a drug.

To the members of our Alumni Association these papers and demonstrations were of particular interest as it showed the value of the laboratory method in teaching medicine and also the splendid selection of such competent teachers as Professors Pratt and Butsch in their respective departments.

Dr. G. H. A. Clowes, Biological Chemist of the New York State Institute for Malignant Diseases, then gave a paper on "Treatment of Hay Fever by Means of a Vaccine Derived From Pollen."

After a luncheon in the College Library, Dr. Wm. House, '95, Professor of Medical Jurisprudence and Assistant Professor of Neurology, Medical Department, University of Oregon, Portland, Oregon, one of our prominent alumni from the west, gave an interesting address on "Apoplexy in Relation to Blood Pressure." He was followed by Dr. Hugh C. McDowell, 1911, who gave an interesting talk on "Medicine in Mexico," the doctor having spent a year there in practice.

Thursday, May 29th, was essentially our big day. We had with us two such noted masters in medicine as Professor Howard A. Kelly of Johns Hopkins Medical School and Richard C. Cabot of the Harvard Medical School. Professor Kelly opened the day's program with a most interesting address on "The Uses of Radium in Surgery." After discussing very completely the physics and chemistry of this most valuable substance he gave an elaborate historical review of its discovery and then told of the difficulties encountered in obtaining it. The doctor told of the many wonderful results from its use in epithelioma of the face, inoperable carcinomas and prophesied that uterine fibroids henceforth would not require any further surgical intervention by means of the knife. Dr. Kelly is now the possessor of $50,000 worth of radium and hopes to procure at least double that amount for use in his cases.

In the short intermission before Doctor Kelly's clinic, Doctor Thomas F. Story, Secretary-General of the Fourth International Congress of School Hygiene, explained the essential purposes of the convention which is to meet in Buffalo August 25-29 of this year. It is hoped that our alumni will be in evidence at this important meeting.

At the Gynecologic Clinic, arranged for by Doctor Goldsborough for Prof. Kelly, three cases of pelvic infection, respectively 24 and 30 years of age, were shown and fully discussed, special emphasis being laid on the symptomatology and physical signs of the respective cases and the advisability of conservative treatment in such cases.

At 1:30 Dean Williams and the Faculty tendered a luncheon to the alumni in the College Library, at which there were by actual count 239 present.

At the afternoon session Prof. Cabot gave a medical clinic in five cases which Dr. DeLancy Rochester arranged for. There were two cases cardio-sclerosis in the uncompensated stage, one case of mitral stenosis in a young girl, one case of congenital heart disease (a patulous ductus arteriosis) in a young boy, and a case of pernicious anemia. This was followed by an address, "Gynecology, Retrospective and Prospective," in which Prof. Kelly, after tracing the beginnings of gynecology down to the present time, gave a very glowing and masterly prophetic outlook for his favorite specialty.

### The Annual Business Meeting.

The annual business meeting of the association, on Wednesday afternoon, was called to order by the acting President, Dr. Wm. R. Palmer of Johnsonburg, Pa., first vice-President of the association. In the routine business that regularly came before the association, the Secretary read the necrology list that showed the death of twenty-nine of our alumni during the last year.

List:

Frederick F. Hoyer, class '49, said to have been the oldest practitioner in New York State, died at his home in Tonawanda, August 16, 1912, aged 90 years.

John G. Collver, class '54, practiced for more than forty years at Waterford, Ont., died September 2, 1912, aged 85 years.

Francis Findlay, class '60, of Franklinville, N. Y., died in Parkville, Mo., October 3, aged 78 years.

Coleman N. Clark, class '65, died January 30, 1913, at St. Charles, Minn., aged 73 years.

Robert A. Gunn, class '66, died at Bellevue Hospital, December 7, aged 68 years.

Ernest L. Shurley, class '66, died at Harper Hospital, Detroit, Mich., of heart disease, May 10, 1913, aged 67 years.

Albert C. Bunn, class '67, first medical missionary to interior of China, died at Asheville, N. C., December 24, 1912, aged 67 years.

Malcolm N. McNaughton, class *68, died at Villisca, Iowa, June 23, aged 63 years.

Frank A. Jones, class '69, died at Rochester, N. Y., aged 53 years.

Wm. Quincy Huggins, class '70, one of the oldest practitioners of Niagara County, died at his home, Sanborn, N. Y., October 21, 1912, aged 70 years.

Ludwig O. Thoma, class '70, died in Berlin Hospital on June 22d.

DeWitt C. Hunter, class '73, practitioner for more than fifty years, died at his home in Rochester, N. Y., October 21, 1912, aged 78 years.

Wm. E. Fitzgibbons, class '74, of Oglesby, Ill., formerly of Wataga, and for two years Health Officer of Peru, died at La Salle, Ill., October 3, 1912, aged 63 years.

Herbert P. Sheldon, class '79, died at his home in Scottsbluff, October 3, aged 57 years.

Pardon L. Kimball, class '80, veteran of Civil War, formerly practitioner in Illinois and Iowa, died at his home in Long Beach, Cal., recently, aged 71 years.

Paul F. Bussman, class '82, died December 19, at his Buffalo home, aged 52 years.

Herbert W. Davis, class '83, died at Jamestown, September 17, aged 50 years.

Delos B. Manchester, class '83, for many years practitioner of Oneonta, N. Y., later resident of Grant's Pass, Oregon, died at the home of his brother at Schenevus, N. Y., aged 54 years.

John W. Burchard, class '84, died at his home in Bradley, S. Dakota, August 15, aged 65 years.

Louis Pfandhoefer, class '86, died at his home, Falls City, Oregon, January 9th, aged 60 years.

Earl A. Scofield, class '87, died at Bemus Point, June 24th, aged 57 years.

Edwin L. Wood, class '88, died at Beausoil, France, June 2d, 1912, aged 51 years.

Alfred W. Henckell, class '89, died at Rochester, N. Y., June 30, 1912, aged 46 years.

Eva Viola Mead, class 1900, died at her home in Buffalo, November 20, 1912, aged 41 years.

Wm. H. Loughhead, class '91, died in hospital at Hornell, after operation September 12, 1912, aged 52 years.

Edward J. Kiepe, class '97, died at Kansas City, November 23, aged 46 years.

Samuel B. Baker, class 1899, died at his home in Rochester, N. Y., May 27th, from heart disease, aged 43 years.

Clarence S. H. Darlington, class '02, of Oklahoma, died in Buffalo, August 18, aged 33 years.

Charles Richards, class 1904, died suddenly at his home in Sturgis, S. Dakota, August 4th, aged 40 years.

Wm. H. Prudden, class '05, of New York City, died at Roosevelt Hospital, July 24, 1912, aged 30 years.

A special eulogy was given on the life of Dr. Shurley by Dr. DeLancey Rochester, and a set of memorial resolutions drafted by a committee consisting of Henry R. Hopkins, '67; Peter W. VanPeyma, '72, and DeLancey Rochester, '84, was unanimously adopted, after which Dr. Hopkins pronounced a eulogy in a memoriam of Frederick F. Hoyer of class of '49, closing with the following poem, which, for effectiveness and solemnity, could not be excelled:

### Over the River.

Over the river they beckon to me,
White, Moore, Hadley, Lea,
Rochester, Eastman, Mason, see,
Over the river they beckon to me.
Loved ones who've crossed to the farther side,
Hoyer, Shurley, Wende, see,
They watch and beckon and wait for me.
Folwell, Phelps, Davidson, see,
Over the river they beckon to me.

They crossed in the twilight gray and cold,
And the pale mist hid them from mortal view;
The gates of the city we could not see.
Over the river—over the river—

King, Gray, Harrington, see,
My brothers stand waiting to welcome me;
Potter, Dayton, Wyckoff, see,
Over the river they beckon to me.

We only know that their barks no more
May sail with us over life's stormy sea.
Daggett, Nichell, O'Brian, see,
Over the river they beckon to me.
Yet, somewhere, I know, on the unseen shore,
They watch and beckon and wait for me.
Hoyer, Wende, Shurley, see,
Over the river they beckon to me.

HENRY REED HOPKINS, '67.

May 28th, 1913.

### Report of Executive Committee.

May 28, 1913.

*To the Members of the Alumni Association, Medical Department, University of Buffalo:*

Fellow Alumni:

At the last annual business meeting of our Association the Executive Committee of 1911-12 recommended the four following resolutions, which were duly adopted:

1. That class reunions be held every five years in place of every ten (10) years, as heretofore.

2. That an Annual Alumni Dinner be the social feature of every annual meeting.

3. That an alumni catalogue be published.

4. That we request the Council of the University of Buffalo for representation on its body, such representation to be elected by our Association.

The import of these resolutions in rousing a spirit of enthusiasm and thereby inspiring our members with loyalty to Alma Mater cannot be overestimated. The efficacy of the first two resolutions was demonstrated by the large attendance of the "reunion classes," and the social success of the last annual dinner.

The Alumni Register is now in process of compilation, and had it not been for the tardy replies of some of our recalcitrant members, it would have been in the hands of our members ere this. We, therefore, strongly urge that those who have not responded will do *so at once,* so that the register may be as complete as possible. (Cards may be procured from Miss Emma L. Chappell). Complete or incomplete, the catalogue will be in your hands by October or before.

The fourth resolution was referred to a special committee, consisting of Doctors Rochester, Allen, Jones and Kauffman, and their report will be submitted at this meeting.

In order that our alumni may be more firmly united and thereby become a factor for good in the development of our Alma Mater, as a high-class medical center for both medical education and medical research, we recommend:

1. That an Alumni Fund be established by our Alumni Association to be subscribed to by our members and others whom we can influence to give to same. This fund to be controlled by the Alumni and the interest only to be used for the purpose or purposes as the members of this Association may direct for the benefit of our Alma Mater, the Medical Department of the University of Buffalo.

2. That branch Alumni Associations with local organizations be established where there are sufficient numbers to warrant the organization.

3. That steps be taken toward the formation of an Associated Alumni of all the departments of the University of Buffalo.

4. In order that these purposes may be the more readily and effectively accomplished, the Committee recommend that our Alumni Association be duly incorporated.

5. We strongly urge the adoption of the recommendation of our efficient Treasurer, Dr. W. F. Jacobs, in regard to the abolition of Life Membership in our Association for the Alumni residing in Buffalo and vicinity.

Respectfully submitted,

LESSER KAUFFMAN, *Chairman,*
HARRY MEAD,
WM. WARD PLUMMER.

The Committee on Conference with the Council of the University relative to granting our Association representation on its body, said representative to be elected by our Association, reported through the Chairman, Dr. DeLancey Rochester, that the Council favored our resolution and were of the opinion that the Association elect a representative for a period of three to four years, said representative to take office on resignation or death in the present Council. On motion of Dr. Grover Wende, amended by Dr. Kauffman, it was unanimously agreed that the Alumni Association approve the action of the Council and elect its representative for a period of four years, said representative serving such time as there may be a vacancy on the Council until commencement 1917, when a successor to the member-elect be chosen. Dr. Peter W. VanPeyma of the class of '72 was then unanimously elected as representative from our association on the Council of the University of Buffalo. It is hoped that this action will tend to arouse a better feeling among our Alumni to our Alma Mater, and that the spirit of loyalty and enthusiasm for Alma Mater will become more markedly in evidence from year to year, and will help to make the medical department one of the leading schools for instruction and research in medicine.

Dr. Herbert U. Williams, Dean of Department of Medicine, made the following statement at our business meeting, which you are requested to read over carefully:

The Executive Committee have asked me to make a statement to the Alumni similar to that I made a year ago. On that occasion the organization and general condition of the Medical Department were discussed, and it will not be necessary to cover that ground again. I wish, however, to repeat one statement made at that time, namely, that the University of Buffalo is not a stock company, and that although it was originally organized as a stock company, the stock was abolished by act of legislature several years ago. The title to the property of the University is vested in the Council .

Probably the most important move that the Medical Department have made during the last year has been to increase the entrance requirements, beginning with September, 1914. After that date every student entering upon the study of medicine will be required to have completed a year of work of college grade in Physics, Chemistry, Biology and either French or German. The Council of the University of Buffalo is now organizing courses in arts and sciences of college grade, equivalent to the freshman year of the B. S. course in standard colleges. During the session of 1913-14 the classes will be held in the medical building. Students intending to study medicine will thus be able to obtain preparation in the University.

The University has a field of work in Buffalo and the surrounding country entirely outside of the teaching in the regular departments. University extension methods have been used very extensively in many parts of the country. A beginning was made by the University of Buffalo last winter in no less than four different directions. A course of evening lectures and demonstrations in Physiology were given by Dr. Pratt to a class of teachers from public schools. Dr. Sy and others gave a course of lectures on Domestic Chemistry on Saturday afternoons which were remarkably successful, the attendance constantly being from 150 to 200. A laboratory course in connection with these lectures was attended by a group of young women. Still another course of lectures was given on Sunday afternoons on a variety of topics relating to medicine and public health of a popular character. The course was planned and arranged by Dr. Lucien Howe, who conducted the first lecture. The attendance was usually from 150 to 200 persons. Finally, members of the Faculty have offered their services in giving lectures to outside organizations on medical topics, both in Buffalo and in surrounding towns. The number of requests for such lectures has been large, and a field of usefulness for the University in this direction seems to be clearly indicated. All of these lines of activity, or similar lines, will be followed out during the ensuing year. Our two new Professors, Dr. Pratt in Physiology and Dr. Butsch in Pharmacology, have been of great assistance in these enterprises, as they have been successful in conducting the work in their own departments.

Officers for ensuing year were elected as follows:

President, Grover W. Wende, '89, Buffalo.

1st Vice-President, George E. Brewer, '84, New York City.

2d Vice-President, George W. Goler, '89, Rochester.

3d Vice-President, Frank W. McGuire, '94, Buffalo.

4th Vice-President, Cynthia W. Stover, '06, Williamsport, Pa.

5th Vice-President, Wm. Irving Thornton, '99, Buffalo.

Secretary, Julius Richter, '04, Buffalo.

Treasurer, William F. Jacobs, '08, Buffalo.

Trustee for 5 years, Henry C. Buswell, '88.

Executive Committee, Lesser Kauffman, Harry Mead, Edith R. Hatch.

### The Annual Dinner.

The annual dinner of our Association, which was the social event of our meeting, occurred on Thursday evening, May 29th, at the Hotel Statler. There were 175 present at the table, and after an excellent repast enjoyed by all the toastmaster of the evening, Dr. DeLancey Rochester, was introduced by Dr. Wm. R. Palmer, who presided. Dr. Rochester acquitted himself in his usual happy and effective manner, introducing each speaker fittingly. Prof. Roswell Park replied on behalf of the Faculty, stating the progress our Alma Mater had made in the past year. Dr. S. V. V. Holmes, in his charming and felicitous style, spoke on "College Spirit." Those who were present surely carried away something of the Williams College enthusiasm. Prof. Howard A. Kelly gave a short toast on "The Ideals of the Profession," laying special emphasis on the moral responsibility of the physician. The principal address of the evening was given by Dr. W. K. Wicks of Syracuse, who came by invitation and gave a clinic, "The Anatomy of Humor," which was quite apropos to a gathering of medical men. Dr. W. H. Johnson, President of the graduating Class, responded on behalf of his classmates, and "Tom" McKee spoke for the Reunion Classes in his usual effective and yet pithy manner. If there were a few more McKees in our Association nothing would be too good for our Alma Mater. The surprise of the evening was the presentation of suitable gifts to Miss Emma L. Chappell, who had resigned from the position of College Secretary and Registrar to the Medical Department after faithfully performing the duties of her office for twenty years. In recognition of her fidelity, devotion and uniform courtesy, the general Faculty, through the Chairman of the Special Committee, Dr. Allen A. Jones, presented her a fine traveling bag and a set of resolutions, and on behalf of the Alumni, Dr. Edith R. Hatch responded likewise. It is to be noted that during her connection with the University, Miss Chappell never by word or action ever offended a student, an alumnus or a member of the Faculty.

Impromptu toasts were responded to by Dr. Dewitt H. Shumay for the "Bachelors" on the Faculty, Dr. Wm. House on "Medicine in the Wild and Woolly West," and Dr. Thomas H. Story on "The Fourth International Congress of Hygiene."

### Fraternity Reunions.

The Fraternity Reunions were held on Wednesday night. The Nu Sigma Nu (I. C. I. Chapter) held a banquet which was very largely attended at the Hotel Statler. The Phi Rho Sigma (Alpha Omega Delta Chapter) and the Omega Upsolon Phi entertained their members at their Chapter Houses.

The attention of the Alumni is directed to the U. of B. paper. *The University Bison,* which is edited and controlled by the student body. This paper is to appear monthly during the college year and contain all the college and departmental news—a special feature is to be the Alumni page. Better subscribe, if you have not done so. Cost 25 cents per annum.

In summing up the results attained during the past year, it is interesting to note a steady and healthy increase in every particular. Registration in 1912, 263; in 1913, 324. In 1912, after refunding the Faculty over $120 for money advanced for printing, etc., we showed a balance of $132. This year our balance is over $250. The annual dinner in 1912 showed an attendance of 130, this year 175. The attendance at the clinics, business meeting and luncheon were all commensurably increased.

The Executive Committee wish to call your attention that the 39th annual meeting occurs June 2-5, 1914; the classes of 1864, 1869, 1874, 1879, 1884, 1889, 1894, 1899, 1904 and 1909 will hold reunions. Arrangements are already under way for a better attendance and a program equally as good as this year. Remember, please, that a new constitution and by-laws are to be presented, as well as action taken on the incorporation of our Association. It therefore behooves every alumnus, even if he or she be not a member of the reunion classes stated above, to make it a point to attend.

For the benefit of the reunion classes, it should be stated that the class of 1904 is already planning its tenth reunion. This class wishes to show its fellow alumni what enthusiasm and loyalty to Alma Mater means.

---

Aleucocythaemic Leukaemia. Rupert Waterhouse, *Bristol Medico-Chir. Jour.*, March, 1913, refers to the literature and cites three cases, the red cells ranging about a million, sometimes less; the whites from 6000 to 7000 in one case, 8600-12800 in another, 3000-108000 in the third. The presence of 90 per cent. or more of lymphocytes, with myelocytes, slight enlargement of the spleen, epistaxis and enlarged glands, especially cervical, he considers adequate to a diagnosis. (Note—Personally, we must confess that, with the part of Hamlet omitted, we should scarcely recognize the play as Hamlet, unless the lines were otherwise very closely followed.)

---

Virginal Mammary Hypertrophy. Battle Malone, *Memphis Med. Mo.*, Dec., 1912, reports a case in a girl of 14, the growth having occurred within a year, from the size of hens' eggs to enormous masses reaching to the pubes. Elevation of the breasts was attempted as a palliative, but always brought on rigors and elevation of temperature to 103 or 104. They were, therefore, removed. Pathologic examination revealed numerous small cysts and increase of fibrous tissue with areas of newly formed glandular cells. Recovery.

---

Transposition of Viscera. S. Neuhof, *Post Graduate*, reports congenital cases in brother and sister. Another sister had cardiac enlargement with mitral and probably pulmonary leakage, following endocarditis, in a heart originally normally placed.

